# Supplementary material for: Causal Relationship between Aging and Anorexia Nervosa: A White-Matter-Microstructure-Mediated Mendelian Randomization Analysis
Source: Biomedicines. 2024 Aug 16;12(8):1874. doi: 10.3390/biomedicines12081874 (PMC11351342; doi:10.3390/biomedicines12081874)
Supplement: Supplementary file 1 [file biomedicines-12-01874-s001.zip › Supplementary Figure/Figure S4/Singe SNP plot of MR analysis for TL to ubm-b-1942.pdf]

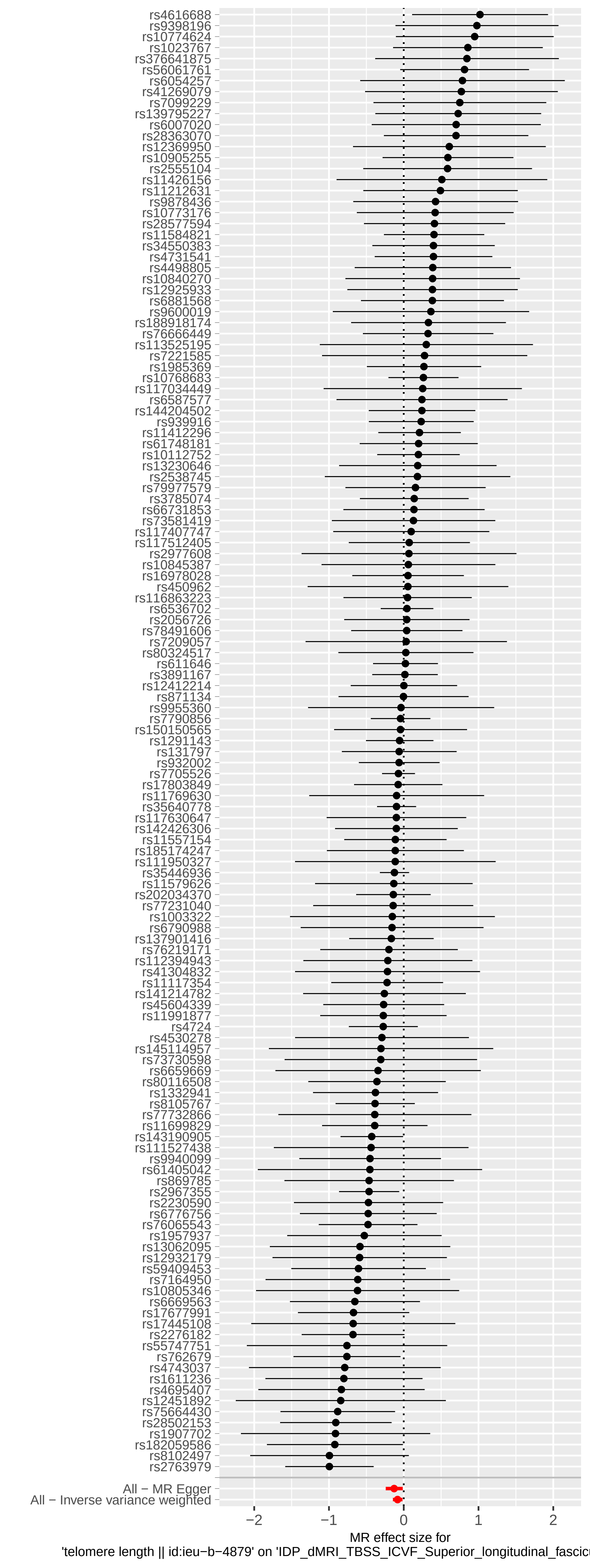
rs4616688
rs9398196
rs10774624
rs1023767
rs376641875
rs56061761
rs6054257
rs41269079
rs7099229
rs139795227
rs6007020
rs28363070
rs12369950
rs10905255
rs2555104
rs11426156
rs11212631
rs9878436
rs10773176
rs28577594
rs11584821
rs34550383
rs4731541
rs4498805
rs10840270
rs12925933
rs6881568
rs9600019
rs188918174
rs76666449
rs113525195
rs7221585
rs1985369
rs10768683
rs117034449
rs6587577
rs144204502
rs939916
rs11412296
rs61748181
rs10112752
rs13230646
rs2538745
rs79977579
rs3785074
rs66731853
rs73581419
rs117407747
rs117512405
rs2977608
rs10845387
rs16978028
rs450962
rs116863223
rs6536702
rs2056726
rs78491606
rs7209057
rs80324517
rs611646
rs3891167
rs12412214
rs871134
rs9955360
rs7790856
rs150150565
rs1291143
rs131797
rs932002
rs7705526
rs17803849
rs11769630
rs35640778
rs117630647
rs142426306
rs11557154
rs185174247
rs111950327
rs35446936
rs11579626
rs202034370
rs77231040
rs1003322
rs6790988
rs137901416
rs76219171
rs112394943
rs41304832
rs11117354
rs141214782
rs45604339
rs11991877
rs4724
rs4530278
rs145114957
rs73730598
rs6659669
rs80116508
rs1332941
rs8105767
rs77732866
rs11699829
rs143190905
rs111527438
rs9940099
rs61405042
rs869785
rs2967355
rs2230590
rs6776756
rs76065543
rs1957937
rs13062095
rs12932179
rs59409453
rs7164950
rs10805346
rs6669563
rs17677991
rs17445108
rs2276182
rs55747751
rs762679
rs4743037
rs1611236
rs4695407
rs12451892
rs75664430
rs28502153
rs1907702
rs182059586
rs8102497
rs2763979
All − MR Egger
All − Inverse variance weighted
−2
−1
0
1
2
MR effect size for
'telomere length || id:ieu−b−4879' on 'IDP_dMRI_TBSS_ICVF_Superior_longitudinal_fascic
